# Expanding the donor pool in kidney transplantation: Should organs with acute kidney injury be accepted?—A retrospective study

**DOI:** 10.1371/journal.pone.0213608

**Published:** 2019-03-13

**Authors:** Katharina Schütte-Nütgen, Markus Finke, Sabrina Ehlert, Gerold Thölking, Hermann Pavenstädt, Barbara Suwelack, Daniel Palmes, Ralf Bahde, Raphael Koch, Stefan Reuter

**Affiliations:** 1 Department of Internal Medicine D, Division of General Internal Medicine, Nephrology and Rheumatology, University Hospital Münster, Münster, Germany; 2 Department of General and Visceral Surgery, University Hospital Münster, Münster, Germany; 3 Institute of Biostatistics and Clinical Research, University Hospital Münster, Münster, Germany; Robert Bosch Krankenhaus, GERMANY

## Abstract

**Background:**

Given the gap between patients in need of a renal transplantation (RTx) and organs available, transplantation centers increasingly accept organs of suboptimal quality, e.g. from donors with acute kidney injury (AKI).

**Methods:**

To determine the outcome of kidney transplants from deceased donors with AKI (defined as ≥ AKIN stage 1), all 107 patients who received a RTx from donors with AKI between August 2004 and July 2014 at our center were compared to their respective consecutively transplanted patients receiving kidneys from donors without AKI. 5-year patient and graft survival, frequencies of delayed graft function (DGF), acute rejections and glomerular filtration rate (eGFR, CKD-EPI) were assessed.

**Results:**

Patient survival was similar in both groups, whereas death-censored and overall graft survival were decreased in AKI kidney recipients. AKI kidney recipients showed higher frequencies of DGF and had a reduced eGFR at 7 days, three months and one and three years after RTx. However, mortality was noticeably lower compared to waiting list candidates. Rejection-free survival was similar between groups.

**Conclusions:**

In our cohort, both short-term and long-term renal function was inferior in recipients of AKI kidneys, while patient survival was similar. Our data indicates that recipients of donor AKI kidneys should be carefully selected and additional factors impairing short- and long-term outcome should be minimized to prevent further deterioration of graft function.

## Introduction

Renal transplantation (RTx) is the number one choice for treating end-stage renal disease (ESRD) yielding a better prognosis and quality of life compared to dialysis [1,2]. Donor organs are rare, for instance in Germany only 1.497 patients received an organ from a deceased donor in 2016 while 7.876 patients were waiting for a suitable organ on 31^st^ December 2016 [3]. Thus, clinicians are confronted with the question under which conditions an offered donor kidney is responsibly acceptable. The establishment of the so-called rescue allocation led to discard rates for donated kidneys of 7.5% [4].

Lately, attention was drawn to deceased donors who experienced acute kidney injury (AKI, as defined by the AKIN) prior to donation [5]. Causes of AKI are different including many reversible conditions [6]. However, it has been shown that AKI translates to a relevant extend to chronic kidney disease (CKD). AKI and CKD are nowadays considered to be interconnected syndromes [7] and a history of AKI may even promote the transition to ESRD [8].

In the context of RTx, contradictory data on the outcomes of kidneys with AKI have been published. There is a series of small studies yielding that despite higher rates of delayed graft function (DGF) in grafts from AKI donors, there is no significant difference in graft function and one-year survival after transplantation between kidneys with and without donor AKI [9,10]. In contrast, a retrospective analysis of the UK Transplant Registry including 11,219 kidneys (1,869 with AKI) reported a slightly lower graft survival of kidneys from donors with AKI along with higher rates of DGF and primary non-function [11]. However, transplantation conditions and guidelines between the UK and Germany are different, with the most prominent difference being the acceptance of donation after circulatory death (DCD).

There is only limited retrospective data on the outcome of kidneys with donor AKI in Germany [12–14]. All studies are small and have several limitations i.e. missing data on AKIN classification, control group without donor AKI or induction therapy.

We herein provide long-term data on the outcome of kidneys with donor AKI from a large German Transplant center reflecting the characteristics of the German transplantation system.

## Patients and methods

### Patients

Prior to analysis, data of all patients were anonymized and de-identified. The local ethics committee (Ethik Kommission der Ärztekammer Westfalen-Lippe und der Medizinischen Fakultät der Westfälischen Wilhelms-Universität, No. 2014-381-f-N) approved the study. Methods in this study were carried out in accordance with the current transplantation guidelines and the Declarations of Istanbul and Helsinki. Written informed consent was given by all participants at the time of transplantation for recording their clinical data.

We retrospectively analyzed all 107 kidney transplant recipients who received a renal allograft from donors with AKI between August 2004 and July 2014 at the University Hospital Münster. We compared them with their respective 107 patients transplanted immediately afterwards and receiving kidneys from donors without AKI. This matching strategy was applied to avoid bias due to changing therapeutic strategies and guidelines over the relatively long study period of 10 years. We used initial creatinine level and ultrasonography findings for decision making whether to accept or discard an organ as these findings are easily available and do not extend the cold ischemia times, unlike biopsy. Machine perfusion was not performed in any of the cases. Recipients were selected according to Eurotransplant allocation criteria. Patients with combined transplants or missing follow-up data were excluded from analysis.

A group of all 703 patients who were on the waiting list at our center from July 2006 up to January 2015 was assessed for survival analysis.

Data were collected from the patients’ files and the Eurotransplant network information system (ENIS).

### Definition of donor AKI

AKI was defined according to the AKIN classification in congruence with the largest analysis currently available [11]. Creatinine and urine output were considered for classification based on information provided by the Eurotransplant donor report/Eurotransplant network information system (ENIS), independent of the cause of AKI.

### Outcome measures

Main outcome measures were patient survival, overall graft survival and death-censored graft survival. Patient survival was defined as time from RTx or day of listing, respectively, to death (from any cause) or last contact for patients alive. Patients on the waiting list were removed from the follow-up on the day they received a kidney transplantation which was considered as a competing event.

Overall graft survival was defined as time from RTx to death (from any cause), graft failure or last contact whatever occurred first. Graft failure was defined as reinitiation of dialysis treatment. Death-censored graft survival was defined as time from RTx to graft failure where death without prior graft failure was regarded as censored. Further outcome parameters were incidence of delayed graft function (DGF, dialysis within the first week after RTx), serum creatinine and eGFR at 7 days, three months and one and three years after transplantation as well as frequency of biopsy-proven acute rejections (BPAR) and rejection-free survival within the first year after RTx.

Whole blood was analyzed for creatinine (enzymatic assay; Creatinine-Pap, Roche Diagnostics, Mannheim, Germany) and renal function was determined by calculating the eGFR using the CKD-EPI equation.

### Statistical analysis

Statistical analysis was performed using IBM SPSS Statistics 25 for Windows (IBM Corporation, Somers, NY, USA). Normally distributed continuous variables are shown as mean ± standard deviation (SD) and not normally distributed continuous variables as median and 1^st^ and 3^rd^ quartiles. Absolute and relative frequencies are given for categorical variables. Groups were compared using Student’s t-test for normally distributed data, Mann–Whitney U test for skewed distributed continuous variables and Fisher's exact test for categorical variables.

Univariable and multivariable binary logistic regression analysis were performed to estimate the probability of DGF. A stepwise forward variable selection procedure (inclusion criteria: P-value of the likelihood ratio test ≤ 0.05) was performed including the following variables: AKIN stage, recipient age and sex, BMI, time on dialysis, prior kidney transplantation ≥ 1, number of HLA mismatches, current ≥ PRA 20%, induction therapy, cold ischemia time, donor age. All selected variables were then included in a final multivariable model. Results are presented as odds ratios (OR) with 95% confidence intervals. Rejection-free survival was estimated by Kaplan-Meier method and groups were compared by log-rank test [15].

Univariable and multivariable linear regressions were performed to determine independent factors influencing allograft function (eGFR) at indicated time points. Variables included were AKIN stage, recipient age and sex, recipient BMI, time on dialysis, prior kidney transplantation, number of HLA mismatches, current ≥ PRA 20%, cold ischemia time, induction therapy, donor age. Results are presented as regression coefficients (β) with 95% confidence intervals.

Survival analyses were based on a maximum follow-up of 5 years after RTx. Patient survival as well as death-censored and overall allograft survival (death was regarded as event) were estimated using Kaplan-Meier method [15], and groups were compared using log-rank tests. Univariable Cox regressions were performed to estimate the effect of donor AKI on the hazard for death and allograft failure [16]. Results are presented as hazard ratios (HR) with 95% confidence interval (95% CI).

No adjustment for multiple testing was performed and all analyses are regarded as explorative.

Exploratory p-values ≤0.05 were considered as statistically noticeable.

## Results

### Demographics

During the study period 796 RTx from deceased donors including 111 organs with AKI from 86 corresponding donors were performed at our center. Four patients were excluded due to combined RTx or missing follow-up data. Except for a higher age in recipients of AKI kidneys, the groups were similar with respect to all analyzed baseline characteristics (Table 1).

**Table 1:** Baseline characteristics of transplant recipients.

	No AKI (n = 107)	AKI (n = 107)	p-value
**Age (years, mean ± SD)**	53.5 ± 14.6	57.5 ± 14.2	0.044^a^
**Male sex, n (%)**	68 (63.6)	69 (64.5)	1.000^b^
**BMI (kg/m**^**2**^**, mean ± SD)**	25.5 ± 4.4	25 ± 4.2	0.977^a^
**Diagnosis of ESRD, n (%)**			0.320^b^
Hypertension	8 (7.9)	12 (12.6)	
Diabetes	7 (6.9)	10 (10.5)	
Polycystic kidney disease	18 (17.8)	9 (9.5)	
Obstructive Nephropathy	5 (5.1)	3 (3.2))	
Glomerulonephritis	36 (35.6)	35 (36.8)	
FSGS	1 (1)	6 (6.3)	
Interstitial Nephritis	9 (8.9)	7 (7.4)	
Vasculitis	1 (1)	1 (1)	
Other	16 (15.8)	12 (12.6)	
**Time on dialysis (years, median (1**^**st**^**, 3**^**rd**^ **quartile))**	6.1 (4.0, 7.9)	5.9 (2.9, 7.9)	0.494^c^
**Urgency code on wait list, n (%)**			0.482^b^
Transplantable	95 (88.8)	99 (92.5)	
Immunized	12 (11.2)	8 (7.5)	
**Allocation system, n (%)**			0.762^b^
ETKAS	73 (68.2)	67 (62.2)	
ESP	27 (25.2)	29 (27.1)	
REAL	1 (0.9)	1 (0.9)	
HU	6 (5.6)	10 (9.3)	
**≥1 prior kidney transplantat, n (%)**	25 (23.4)	17 (16)	0.228^b^
**Number HLA mismatch, n (%)**			0.552^b^
0–3	77 (72)	72 (67.3)	
4–6	30 (28)	35 (32.7)	
**Current PRA, n (%)**			0.284^b^
0–20%	97 (90.7)	102 (95.3)	
>20%	10 (9.3)	5 (4.7)	
**Induction, n (%)**			1.000^b^
Basiliximab	99 (92.5)	98 (91.6)	
Thymoglobulin	8 (7.5)	9 (8.4)	
**Cold ischemia time (hours, mean ± SD)**	11.9 ± 4.2	10.9 ± 3.9	0.076^a^
**Warm ischemia time (min, mean ± SD)**	31.6 ± 6.8	32.5 ± 6.7	0.315^a^

Demographic characteristics of the study population by donor AKI status. Results are presented as mean ± standard deviation (SD) or median and 1^st^ and 3^rd^ quartile, respectively, or as absolute and relative frequencies.

BMI = body mass index, ESRD = end-stage renal disease, FSGS = focal segmental glomerulosclerosis, ETKAS = EuroTransplant Kidney Allocation System, ESP = European Senior Program, REAL = Recipient Extended Allocation, RA = Rescue Allocation, HLA = human leukocyte antigen, PRA = panel reactive antibodies.

^a^ Student’s t-test

^b^ Fisher’s exact test

^c^ Mann-Whitney U test.

Donor characteristics are shown in Table 2. There were no differences between donors with or without AKI. 84.1% (90) of donor kidneys with AKI were classified as AKIN 1, 12.1% (13) were classified as AKIN 2 and 3.8% (4) as AKIN 3, respectively.

**Table 2:** Donor characteristics.

	No AKI (n = 107)	AKI (n = 107)	p-value
**Age (years, mean ± SD)**	51.1 ± 16.5	54.3 ± 17.2	0.167^a^
**Male sex, n (%)**	60 (56.1)	63 (58.9)	0.782^b^
**BMI (kg/m**^**2**^**, mean ± SD)**	25.4 ± 4	26.7 ± 5.4	0.056^a^
**Donor hypertension, n (%)**	33 (67.3)	39 (69.6)	0.836^b^
**Donor diabetes, n (%)**	7 (21.9)	10 (31.3)	0.572^b^
**Donor nicotine abuse, n (%)**	34 (41.0)	43 (47.8)	0.444^b^
**Donor alcohol abuse, n (%)**	11 (12.5)	20 (21.1)	0.167^b^
**Donor arterial occlusive disease, n (%)**	11 (10.6)	19 (17.8)	0.168^b^
**Donor cardiopulmonal resuscitation (CPR), n (%)**	28 (26.2)	27 (25.2)	1.000^b^
**Donor serum creatinine, mg/dl (median, (1**^**st**^**, 3**^**rd**^ **quartile))**	0.90 (0.70, 1.09)	1.0 (0.80, 1.20)	0.050^c^
**Donor peak serum creatinine, mg/dl (median (1**^**st**^**, 3**^**rd**^ **quartile))**	0.94 (0.76, 1.20)	1.66 (1.30, 2.01)	<0.001^c^
**AKIN stage, n (%)**			-
AKIN I	-	90 (84.1)	
AKIN II	-	13 (12.1)	
AKIN III	-	4 (3.7)	

Demographic characteristics kidney donors by donor AKI status. Results are presented as mean ± standard deviation (SD) or median and 1^st^ and 3^rd^ quartile, respectively, or as absolute and relative frequencies.

BMI = body mass index, CPR = cardiopulmonary resuscitation.

^a^ Student’s t-test

^b^ Fisher’s exact test

^c^ Mann-Whitney U test.

### Main clinical outcomes

**Fig 1:**
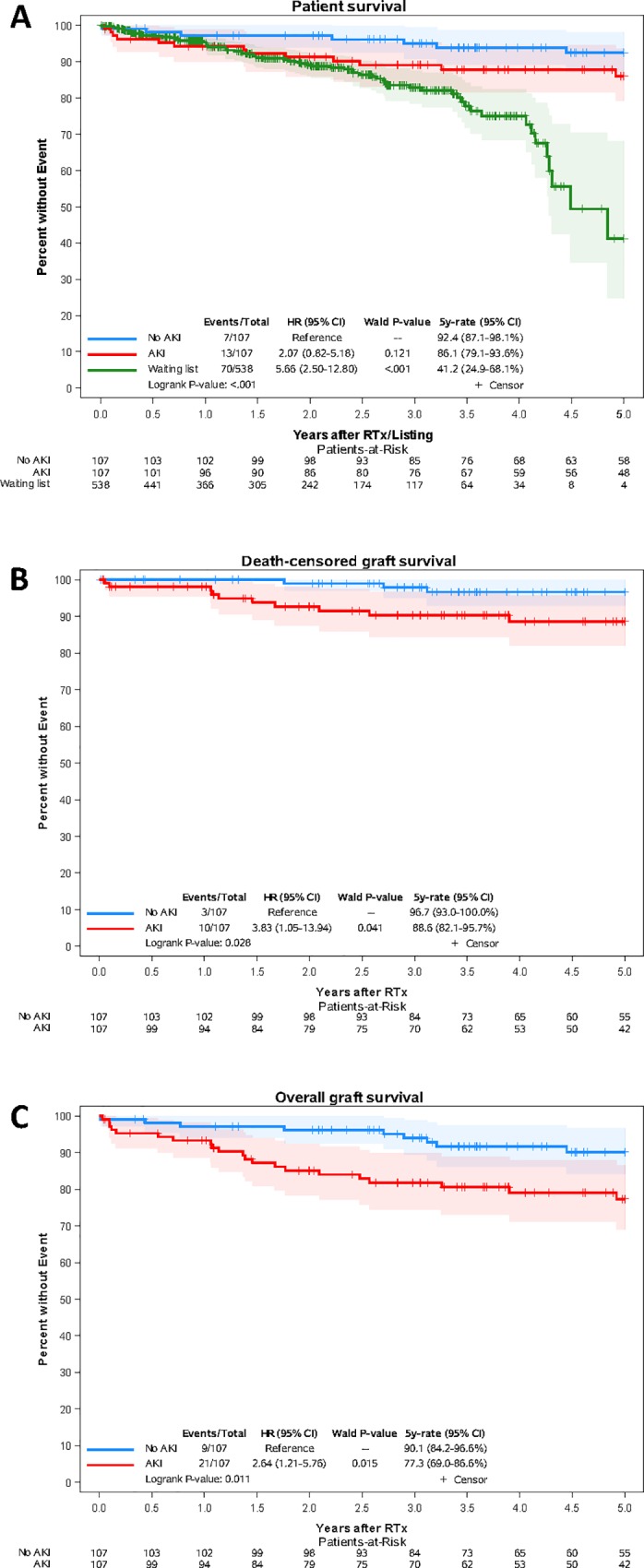
**Kaplan-Meier curves for patient (A), death-censored (B) and overall graft survival (C).** Survival curves and 5-year rates were estimated by Kaplan–Meier method and compared by log-rank test. Band plots represent 95% confidence intervals (log transformation) for pointwise Kaplan-Meier survival estimates. Hazard ratios (HR), 95% confidence limits (CI) and Wald p-values are from univariable Cox-regression. While patient survival was comparable between recipients who received an organ from donors with AKI (red lines) and without AKI (blue lines) (log-rank p = 0.133), patients who remained on the waiting list showed a noticeably reduced survival (log-rank p<0.001 vs. recipients of non-AKI kidneys and log-rank p = 0.001 vs. recipients of AKI kidneys, respectively). Death-censored and overall graft survival was reduced in recipients of AKI kidneys compared to recipients of non-AKI kidneys (log-rank p = 0.028 and 0.011. respectively). Univariable Cox-regression revealed a noticeable association between donor AKI and death-censored and overall graft survival.

Estimated 5-year patient survival was similar between recipients of AKI and non-AKI kidneys (92.4% and 86.1%, respectively, log-rank p = 0.133), whereas patients remaining on the waiting list showed a noticeably lower 5-year patient survival (41.2%) compared to recipients of non-AKI (log-rank p<0.001) and AKI kidneys (log-rank p = 0.011) (Fig 1A). 5-year death-censored and overall allograft survival were noticeably reduced in recipients of AKI donors compared to recipients of donors without AKI (88.6% vs. 96.7%, log-rank p = 0.028 and 77.3% vs. 90.1%, log-rank p = 0.011, respectively) (Fig 1B and 1C). Cox-regression revealed a noticeable association between donor AKI and death-censored (HR 3.83 (95% CI 1.05–13.94), p = 0.041) and overall allograft survival (HR 2.64 (95% CI 1.21–5.76, p = 0.015). Donor age also turned out to be noticeably associated with death-censored and overall graft survival in univariable Cox-regression analysis (S1 Table). Of note, subanalysis of AKI kidney recipients revealed a noticeably reduced patient and overall graft survival in recipients with DGF compared to recipients without DGF (S1 Fig).

### Further clinical outcomes

Recipients of donors with AKIN stages 1 and 2 had higher frequencies of DGF (n = 31 (34.4%) and n = 4 (30.8%), respectively) when compared to non-AKI recipients (n = 20 (18.7%)). While DGF frequency did not differ between patients with AKIN stage 1 and 2 kidneys, DGF occurred much more frequent in kidneys with AKIN stage 3 with three out of four patients experiencing a DGF. Due to the small numbers of events in some AKI stages, we aggregated these data and compared donors with any AKI and without AKI.

DGF occurred more frequently in AKI kidney recipients than in non-AKI kidney recipients in both univariable (S2 Table) and multivariable logistic regression analysis (OR 2.40 (95% CI 1.30–4.48), p = 0.006 and 3.04 (1.56–5.95), p = 0.001, respectively). Other factors associated with DGF in multivariable analysis were prior RTx and number of HLA mismatches (Table 3).

**Table 3:** Multivariable logistic regression model for DGF after variable selection.

Independent variables	OR (95%—CI)	p-value
Intercept	0.21	<0.001
Donor AKI Yes vs. no (ref.)	3.15 (1.59–6.24)	0.001
Prior kidney transplantation ≥ 1 vs. 0 (ref.)	3.27 (1.52–7.07)	0.003
Number HLA mismatch 4–6 vs. 0–3 (ref.)	3.96 (1.70–9.23)	0.001

Besides donor AKI, prior kidney transplantation and a higher number of HLA mismatch turned out to be associated with DGF.

DGF = Delayed graft function, OR = Odds ratio, CI = Confidence interval, ref = reference. P-values are from the Wald tests.

Allograft function was investigated at 7 days, three months and one and three years post transplantation. Patients who received an organ with AKI showed a decreased eGFR at all investigated time points after transplantation (S3 Table). Donor AKI was noticeably associated with a lower eGFR at all times in both univariable and multivariable linear regression. Other variables that were associated with lower eGFR were recipient BMI and donor age (Table 4). A subgroup analysis of the AKI group showed that patients with DGF had a noticeable lower eGFR at 90 days and one and three years compared to patients without DGF (S4 Table). In contrast in recipients of non-AKI kidneys, DGF did not affect eGFR at all investigated time points (S5 Table).

**Table 4:** Multivariable linear regression analysis for eGFR (ml/min/1.73m^2^) at 7 days, three months and one and three years post RTx (n = 210).

	eGFR at 7 days after RTx (n = 210)		eGFR at 3 months after RTx (n = 187)		eGFR at 1 year after RTx (n = 185)		eGFR at 3 years after RTx (n = 157)	
**Independent variables**	**β (95% CI)**	**p-value**	**β (95% CI)**	**p-value**	**β (95% CI)**	**p-value**	**β (95% CI)**	**p-value**
Intercept	120.4 (91.1 to 149.8)	<0.001	116 (92.8 to 139.7)	<0.001	126.2 (101.2 to 151.3)	0.000	122.1 (93.9 to 150.3)	0.000
Donor AKI Yes vs. No (ref.)	-11.2 (-18.3 to -4.2)	**0.002**	-9.3 (-14.6 to -3.9)	**0.001**	-7.9 (-13.6 to -2.1)	**0.008**	-7.4 (-14.0 to -0.8)	**0.029**
Recipient age (x vs. X-1 years)	0.2 (-0.1 to 0.5)	0.193	0.1 (-0.2 to 0.3)	0.572	-0.1 (-0.4 to 0.1)	0.346	-1.8 (-0.5 to 1.0)	0.193
Recipient gender Male vs. female (ref.)	1.6 (-5.9 to 9.1)	0.682	2.6 (-3.0 to 8.3)	0.356	2.1 (-4.0 to 8.1)	0.500	-4.8 (-12.0 to 2.4)	0.193
Recipient BMI (x vs. x-1 kg/m^2^)	-1.9 (-2.8 to -1.0)	**<0.001**	-1.4 (-2.1 to -0.7)	**<0.001**	-1.4 (-2.1 to -0.7)	**<0.001**	-1.2 (-2.0 to 3.2)	**0.007**
Time on dialysis (x vs. x-1 years)	-0.7 (-2.0 to 0.6)	0.314	-0.4 (-1.4 to 0.6)	0.466	-0.3 (-1.7 to 0.8)	0.580	0.0 (-0.1 to 0.1)	0.586
Prior kidney transplantation ≥ 1 vs. 0 (ref.)	-9.5 (-19.1 to 0.1)	0.052	-7.1 (-14.3 to 0.0)	0.051	-2.3 (-10.2 to 5.6)	0.567	-1.3 (-10.2 to 7.6)	0.767
Number HLA mismatch 4–6 vs. 0–3 (ref.)	1.6 (-6.7 to 9.9)	0.707	2.8 (-3.3 to 8.9)	0.360	1.5 (-5.1 to 8.1)	0.648	0.0 (-7.8 to 7.9)	0.996
Current PRA % > 20% vs. ≤ 20% (ref.)	2.6 (-12.5 to 17.7)	0.738	4.6 (-6.4 to 15.6)	0.414	-0.3 (-12.8 to 12.1)	0.962	-3.6 (-21.6 to 14.3)	0.689
Induction Thymoglobin vs. Basiliximab (ref.)	-6.5 (-19.6 to 6.6)	0.328	-1.5 (-11.0 to 8.0)	0.756	-8.0 (-19.0 to 3.0)	0.155	-3.0 (-15.6 to 9.7)	0.643
Cold ischemia time (x vs. x-1 hours)	-1.3 (-2.2 to -0.4)	**0.005**	-0.2 (-0.9 to 0.5)	0.488	0.0 (-0.8 to 0.7)	0.946	-0.3 (-1.1 to 0.5)	0.483
Donor age (x vs. x-1 years)	-0.5 (-0.8 to -0.3)	**<0.001**	-0.5 (-0.7 to -0.3)	**<0.001**	-0.6 (-0.8 to -0.3)	**<0.001**	-0.4 (-0.7 to -0.2)	**0.001**

Besides donor AKI, a higher recipient BMI and donor age are associated with eGFR decline after RTx.

β = regression coefficient, CI = Confidence interval. P-values are from the Wald tests.

Rejection-free survival within one year after transplantation was similar between recipients of grafts with and without AKI (79.4% and 82.2%, respectively, log-rank p = 0.627).

## Discussion

The gap between organ supply and demand in kidney transplantation has led to different strategies to reasonably enlarge the donor pool.

Some of the approaches aim at an expansion of the living donor pool: While ABO-incompatible transplantation is established in the clinical routine of many transplantation centers, HLA-incompatible transplantation is expected to become increasingly important following data from a large study showing a clear survival benefit in these patients [17,18]. Until today, paired kidney exchange is not routinely performed in Germany due to legal uncertainties.

At present, over 70% of all kidney transplantations in Germany utilize kidneys from a deceased donor [3]. Ideally, all of these organs would be of young age, have a normal function and be geographically located nearby, so that short ischemia times could be guaranteed. Unfortunately, the disparity between available organs and the number of patients on the waiting list does not allow the selection criteria to be too strict. For a maximal utilization of the donor pool, transplant centers increasingly accept organs with suboptimal quality, e.g. from donors with AKI.

So far, long-term data on the outcome of AKI kidneys is limited. Defined standard criteria to estimate the quality of kidneys with AKI are still missing. To our knowledge, we herein present the largest 5-year follow-up of patients with AKI kidneys from a large German Transplant center reflecting the characteristics of the German transplantation system. The aim of our study was to assist on-call clinicians in their decision-making to accept or decline kidneys form donors with AKI.

Our and other German data suggest that AKI kidneys are currently used with caution: Most transplanted AKI kidneys were staged as AKIN 1, only a few AKIN 3 kidneys were regarded as suitable for surgery.

Though recent studies on the outcome of kidneys with donor AKI did not show any difference in patient survival, results regarding transplant survival are heterogeneous. Several small studies including the German ones report similar graft and patient survival rates after transplantation despite consistently higher rates of DGF in recipients of donor AKI kidneys [10,12–14,19–22]. However, most studies included only a small number of patients or had a relatively short follow-up period. In contrast, Boffa et al. observed a small difference in 3-year and 5-year graft survival in a larger cohort of British patients. Unfortunately, their long-term follow-up data based on their registry study were only complete for half of the patients [11]. In congruence to these data, we observed no noticeable difference in 5-year patient survival and a lower 5-year transplant survival in patients receiving an AKI kidney. Of note, both, AKI and non-AKI kidney recipients showed a noticeable higher patient survival rate compared to patients remaining on the waiting list. Recipients of AKI kidneys were older than patients receiving non-AKI kidneys (53.5 vs. 57.5 years, p = 0.04). In a study from Essen (Germany), higher recipient age (cutoff was 56 years) was identified as a predictor for lower patient survival in recipients of kidneys from deceased donors with elevated serum creatinine while the influence of recipient age on graft survival was not assessed [14]. Older patients are obviously more prone to die sooner. However, there are contradictory studies on the influence of recipient age on graft survival. While Abou-Jaoude et al. and Al-Shraideh et al. did not find any influence of recipient age on death-censored graft survival and allograft function [23,24], Neri and colleagues report a decreased death-censored allograft survival in older patients [25]. A survey analyzing data from the US Renal Data System (USRDS) between 1988 and 1997 revealed recipient age to be independently associated to an increased graft loss [26]. However, in an analysis on the OPTN database from 1995 to 2000, the results were exactly opposite showing an increased death-censored graft survival with the recipient age [27]. In most studies elderly patients showed diminished patient and graft survival, while death-censored graft survival rates were comparable to younger patients because the major cause of graft failure for the geriatric population seemed to be death with functioning graft.

We herein separately assessed patient survival, death-censored and overall graft survival. In univariable Cox-regression, the recipient age was not associated with death-censored graft survival. Moreover, multivariable analysis for eGFR did not reveal a significant influence of recipient age on kidney function at any of the investigated time points post transplantation, which strongly suggests that the different outcomes are not driven by a difference in the mean age between the groups. As patient survival was similar between the groups, it makes it even more unlikely that the difference in recipient age might have contributed to the different outcomes, suggesting a negligible impact of potential age-related factors as disease burden, functional status or comorbidities in our cohort. The similar rejection-free survival rates as well as comparable frequencies of the respective allocation system between the groups also makes it unlikely that an age-related higher susceptibility to acute rejections or allocation of lower quality organs to elderly patients might have contributed to the difference in allograft function and survival in the present study.

In congruence to the literature, we observed higher rates of DGF in donor kidneys with AKI. DGF is usually recognized to limit function as well as the prognosis of graft and recipients [28,29]. Interestingly, subgroup analysis of recipients with AKI showed a noticeable influence of DGF on patient and overall graft survival as well as on eGFR, while DGF did not affect the outcome in recipients of non-AKI kidneys. These results underline the necessity to prevent DGF and to carefully select patients who are designated to receive a graft with a high probability to develop DGF.

Many studies observed that a higher percentage of DGF in the AKI groups did not lead to a clinically relevant decrease in 6-month or 12-month eGFR [11,30]. This is somehow surprising, as patients with DGF are known to have a higher creatinine in follow-up compared to those without DGF. Hall et al. even reported that allograft function was progressively better for recipients with DGF who received kidneys from donors with increasing stages of AKI [30]. Our data did not match these results, as we observed a difference in eGFR between the two groups of approximately 10 ml/min/1.73m^2^ at three months, one and three years after transplantation. Furthermore, in donor AKI recipients we observed a noticeable influence of DGF on short- and long-term kidney function. One theoretical reason for this finding could be a higher rate of expanded criteria donors (ECDs) in our AKI group, which have been shown to be associated with a worse outcome compared to standard criteria donors [31], especially in recipients with donor AKI [13]. As we found no differences in age or vascular comorbidities in donors with or without AKI, this is, however, unlikely.

Further covariables that were independently associated with DGF and lower eGFR were prior RTx and number of HLA mismatches and recipient BMI and donor age, respectively. This is in accordance with previous studies reporting higher rates of DGF and impaired graft function in presensitized patients [29,32]. Based on this, induction therapy with thymoglobin might be of advantage as shown by Charpentier et al. [33] to prevent DGF. However, the current Cochrane analysis did not reveal any significant effect of thymoglobin induction on DGF though patients with donor AKI and/or prior RTx or higher number of HLA mismatches were not investigated separately [34]. In our study induction therapy was similar between groups, but as choice of thymoglobin induction is normally related to a higher pretransplant patient and donor risk profile, this might mitigate a potential positive effect of thymoglobin induction in our patients.

There is comprehensive literature confirming the influence of early adverse events on long-term allograft outcome. However, it has recently been suggested by Gaston et al. in a prospective multicenter cohort study that late graft failure is not necessarily dependent on pretransplant or early events initiating an inalterable sequence of fibrosis and atrophy, designated as the non-specific diagnosis of chronic allograft nephropathy. It rather represents the real-time impact of new onset graft dysfunction beyond 90 days, regardless of cause, that occurs over the life of an allograft [35]. In the present study both, DGF as well as inferior graft function in the longer term at three months, one and three years, might have contributed to the reduced graft survival of kidneys from donors with AKI. In summary, when considering the transplantation of donor AKI kidneys, additional risk factors–both early and later events—that can impair renal graft function should be taken into account to reduce the risk for graft failure to a minimum.

Our study has some limitations. Data was analyzed retrospectively and all inferential statistics have to be interpreted exploratory. Due to the sample size and the small number of events (patient and graft death), the inclusion of all clinically relevant variables in a multivariable model was not possible to reduce further potential bias. No sensitivity analysis or validation of the selected multivariable models was performed. Therefore, the applied statistical models are not valid to prove associations between potential predictors and the corresponding outcomes. Consequently, prospective studies are needed to confirm our findings.

### Conclusion

Although we have found a reduced graft survival of AKI kidneys, we would like to highlight a few things: We could observe that patient survival between AKI and non-AKI recipients did not differ and was noticeably better compared to patients remaining on dialysis. Therefore, organs with AKI should not be simply discarded. However, as DGF rates are high and prognosis is limited the recipient should be carefully selected and additional covariables that can contribute to DGF or inferior graft function in the short- as well as long-term course (e.g. immunized patients, recipient BMI and donor age as observed in the present study) should be considered.

## Supporting information

S1 Fig **Kaplan-Meier curves for patient (A), death-censored (B) and overall graft survival (C) in recipients of AKI kidney by DGF status.** Survival curves and 5-year rates of AKI kidney recipients with DGF (red lines) and without DGF (blue lines) were estimated by Kaplan–Meier method and compared by log-rank test. Band plots represent 95% confidence intervals (log transformation) for pointwise Kaplan-Meier survival estimates. Hazard ratios (HR), 95% confidence limits (CI) and Wald p-values are from univariable Cox-regression. Patients with DGF showed a noticeable reduced patient and overall graft survival (log-rank p = 0.024 and log-rank p = 0.030, respectively). Univariable Cox-regression revealed a noticeable association between DGF and patient and overall graft survival.
(DOCX)

S1 Table Univariable Cox regression analyses for death-censored and overall graft survival (n = 214). Besides donor AKI, donor age was noticeably associated with death-censored and overall graft survival.
HR = Hazard ratio, CI = Confidence interval, ref = reference. P-values are from the Wald tests.
(DOCX)

S2 Table Univariable logistic regression model for influence on DGF. Additionally, a multivariable model with stepwise forward variable selection procedure was performed. All variables with p ≤ 0.05 (Likelihood ratio test) were included in the final multivariable model (Table 3).
R = Odds ratio, CI = Confidence interval, ref = reference, P-values are from the Wald tests.
(DOCX)

S3 Table eGFR (CKD-EPI, ml/min/1.73m2, median (1st, 3rd quartile)) at 7 days, three months and one and three years post RTx. Recipients of kidneys from donors with AKI show a noticeably decreased eGFR at all times. P-values are from Mann-Whitney U tests.
(DOCX)

S4 Table eGFR (CKD-EPI, ml/min/1.73m2, median (1st, 3rd quartile)) at three months, one and three years post RTx in recipients with AKI. Patients with DGF show a noticeably decreased eGFR at all times. P-values are from Mann-Whitney U tests.
(DOCX)

S5 Table eGFR (CKD-EPI, ml/min/1.73m2, median (1st, 3rd quartile)) at three months, one and three years post RTx in recipients without AKI. Patients with DGF and no DGF show a comparable eGFR at all time points. P-values are from Mann-Whitney U tests.
(DOCX)
