# Supplementary material for: Expanding the donor pool in kidney transplantation: Should organs with acute kidney injury be accepted?—A retrospective study
Source: PLoS One. 2019 Mar 13;14(3):e0213608. doi: 10.1371/journal.pone.0213608 (PMC6415810; doi:10.1371/journal.pone.0213608)
Supplement: S1 Fig — Kaplan-Meier curves for patient (A), death-censored (B) and overall graft survival (C) in recipients of AKI kidney by DGF status. Survival curves and 5-year rates of AKI kidney recipients with DGF (red lines) and without DGF (blue lines) were estimated by Kaplan–Meier method and compared by log-rank test. Band plots represent 95% confidence intervals (log transformation) for pointwise Kaplan-Meier survival estimates. Hazard ratios (HR), 95% confidence limits (CI) and Wald p-values are from univariable Cox-regression. Patients with DGF showed a noticeable reduced patient and overall graft survival (log-rank p = 0.024 and log-rank p = 0.030, respectively). Univariable Cox-regression revealed a noticeable association between DGF and patient and overall graft survival. (DOCX) [file pone.0213608.s001.docx]

**Supporting information**

**Supplemental Figure 1**

**A**

**B**

**C**

**S1 Figure.** **Kaplan-Meier curves for patient (A), death-censored (B) and overall graft survival (C) in recipients of AKI kidney by DGF status.** Survival curves and 5-year rates of AKI kidney recipients with DGF (red lines) and without DGF (blue lines) were estimated by Kaplan–Meier method and compared by log-rank test. Band plots represent 95% confidence intervals (log transformation) for pointwise Kaplan-Meier survival estimates. Hazard ratios (HR), 95% confidence limits (CI) and Wald p-values are from univariable Cox-regression. Patients with DGF showed a noticeable reduced patient and overall graft survival (log-rank p=0.024 and log-rank p=0.030, respectively). Univariable Cox-regression revealed a noticeable association between DGF and patient and overall graft survival.
