# Supplementary material for: Expanding the donor pool in kidney transplantation: Should organs with acute kidney injury be accepted?—A retrospective study
Source: PLoS One. 2019 Mar 13;14(3):e0213608. doi: 10.1371/journal.pone.0213608 (PMC6415810; doi:10.1371/journal.pone.0213608)
Supplement: S1 Table — Besides donor AKI, donor age was noticeably associated with death-censored and overall graft survival. HR = Hazard ratio, CI = Confidence interval, ref = reference. P-values are from the Wald tests. (DOCX) [file pone.0213608.s002.docx]

**Supporting information**

|  | **Death-censored graft survival** | | **Overall graft survival** | |
| --- | --- | --- | --- | --- |
| **Independent variables** | **HR (95% CI)** | **p-value** | **HR (95% CI)** | **p-value** |
| Donor AKI  Yes vs. no (ref.) | 3.83 (1.06 – 13.94) | ***0.041*** | 2.64 (1.21 – 5.77) | ***0.015*** |
| Recipient age (years) | 1.04 (0.99 – 1.09) | 0.122 | 1.03 (1.00 – 1.06) | ***0.042*** |
| Recipient gender  Male vs. female (ref.) | 0.87 (0.28 – 2.64) | 0.799 | 1.08 (0.50 – 2.32) | 0.835 |
| Recipient BMI (kg/m^2^) | 1.01 (0.89 – 1.15) | 0.885 | 1.00 (0.92 – 1.09) | 0.957 |
| Time on dialysis (years) | 0.84 (0.68 – 1.03) | 0.087 | 0.90 (0.79 – 1.03) | 0.126 |
| Prior kidney transplantation  ≥ 1 vs. 0 (ref.) | 1.25 (0.35 – 4.55) | 0.732 | 1.28 (0.55 – 2.97) | 0.572 |
| Number HLA mismatch  4-6 vs. 0-3 (ref.) | 0.74 (0.20 – 2.68) | 0.644 | 2.13 (1.04 – 4.36) | ***0.039*** |
| Current PRA %  > 20% vs. ≤ 20% (ref.) | 1.32 (0.17 – 10.16) | 0.791 | 1.80 (0.54 – 5.91) | 0.340 |
| Induction  Thymoglobin vs. Basiliximab (ref.) | 1.06 (0.14 – 8.15) | 0.956 | 1.94 (0.68 – 5.56) | 0.218 |
| Cold ischemia time (hours) | 0.92 (0.79 – 1.07) | 0.262 | 0.94 (0.86 – 1.04) | 0.214 |
| Donor age (years) | 1.07 (1.02 – 1.12) | ***0.004*** | 1.03 (1.01 – 1.06) | ***0.014*** |

**S1 Table. Univariable Cox regression analyses for death-censored and overall graft survival (n=214).** Besides donor AKI, donor age was noticeably associated with death-censored and overall graft survival.

HR = Hazard ratio, CI = Confidence interval, ref = reference. P-values are from the Wald tests.
