# Supplementary material for: Expanding the donor pool in kidney transplantation: Should organs with acute kidney injury be accepted?—A retrospective study
Source: PLoS One. 2019 Mar 13;14(3):e0213608. doi: 10.1371/journal.pone.0213608 (PMC6415810; doi:10.1371/journal.pone.0213608)
Supplement: S2 Table — Additionally, a multivariable model with stepwise forward variable selection procedure was performed. All variables with p ≤ 0.05 (Likelihood ratio test) were included in the final multivariable model (Table 3). R = Odds ratio, CI = Confidence interval, ref = reference, P-values are from the Wald tests. (DOCX) [file pone.0213608.s003.docx]

**Supporting information**

| **Independent variables** | **OR (95% - CI)** | **p-value** |
| --- | --- | --- |
| Donor AKI  Yes vs. no (ref.) | 2.40 (1.29 – 4.48) | ***0.006*** |
| Recipient age (years) | 0.98 (0.96 – 1.00) | ***0.041*** |
| Recipient sex  Male vs female (ref.) | 1.22 (0.64 – 2.30) | 0.550 |
| Recipient BMI (kg/m^2^) | 1.02 (0.95 – 1.10) | 0.534 |
| Time on dialysis (years) | 1.05 (0.94 – 1.17) | 0.395 |
| Prior kidney transplantation  ≥ 1 vs. 0 (ref.) | 2.80 (1.38 – 5.66) | ***0.004*** |
| Number HLA mismatch  4-6 vs. 0-3 (ref.) | 3.608 (1.59 – 8.13) | ***0.002*** |
| Current PRA %  > 20% vs. ≤ 20% (ref.) | 2.54 (0.88 – 7.35) | 0.086 |
| Induction  Thymoglobin vs. Basiliximab (ref.) | 2.61 (0.96 – 7.14) | 0.061 |
| Cold ischemia time (hours) | 1.05 (0.97 – 1.12) | 0.238 |
| Donor age (years) | 0.99 (0.97 – 1.01) | 0.299 |
| Donor history of hypertension  Yes vs. no (ref.) | 1.10 (0.44 – 2.75) | 0.842 |

**S2 Table. Univariable logistic regression model for influence on DGF.** Additionally, a multivariable model with stepwise forward variable selection procedure was performed. All variables with p ≤ 0.05 (Likelihood ratio test) were included in the final multivariable model (Table 3).

OR = Odds ratio, CI = Confidence interval, ref = reference, P-values are from the Wald tests.
