# Supplementary material for: Expanding the donor pool in kidney transplantation: Should organs with acute kidney injury be accepted?—A retrospective study
Source: PLoS One. 2019 Mar 13;14(3):e0213608. doi: 10.1371/journal.pone.0213608 (PMC6415810; doi:10.1371/journal.pone.0213608)
Supplement: S3 Table — Recipients of kidneys from donors with AKI show a noticeably decreased eGFR at all times. P-values are from Mann-Whitney U tests. (DOCX) [file pone.0213608.s004.docx]

**Supporting information**

|  | **No AKI** | **AKI** | **p-value** |
| --- | --- | --- | --- |
| **7 days** | 28.6 (11.8, 47.3) | 13.7 (7.4, 39.6) | 0.002 |
| **3 months** | 50.2 (37.1, 67.0) | 39.7 (28.5, 50.5) | < 0.001 |
| **1 year** | 52.4 (37.1, 70.5) | 42.7 (30.4, 58.2) | 0.003 |
| **3 years** | 53.0 (37.6, 69.1) | 42.7 (34.2, 59.0) | 0.042 |

**S3 Table.** **eGFR (CKD-EPI, ml/min/1.73m2, median (1st, 3rd quartile)) at 7 days, three months and one and three years post RTx.** Recipients of kidneys from donors with AKI show a noticeably decreased eGFR at all times. P-values are from Mann-Whitney U tests.
