# Supplementary material for: Expanding the donor pool in kidney transplantation: Should organs with acute kidney injury be accepted?—A retrospective study
Source: PLoS One. 2019 Mar 13;14(3):e0213608. doi: 10.1371/journal.pone.0213608 (PMC6415810; doi:10.1371/journal.pone.0213608)
Supplement: S4 Table — Patients with DGF show a noticeably decreased eGFR at all times. P-values are from Mann-Whitney U tests. (DOCX) [file pone.0213608.s005.docx]

**Supporting information**

|  | **No DGF** | **DGF** | **p-value** |
| --- | --- | --- | --- |
| **3 months** | 43.7 (37.0, 55.4) | 27.1 (17.4, 34.1) | <0.001 |
| **1 year** | 47.3 (37.3, 61.1) | 34.4 (20.2, 42.3) | 0.001 |
| **3 years** | 44.4 (37.1, 62.2) | 37.5 (26.0, 46.7) | 0.018 |

**S4 Table. eGFR (CKD-EPI, ml/min/1.73m2, median (1st, 3rd quartile)) at three months, one and three years post RTx in recipients with AKI.** Patients with DGF show a noticeably decreased eGFR. P-values are from Mann-Whitney U tests.
