# Supplementary material for: Expanding the donor pool in kidney transplantation: Should organs with acute kidney injury be accepted?—A retrospective study
Source: PLoS One. 2019 Mar 13;14(3):e0213608. doi: 10.1371/journal.pone.0213608 (PMC6415810; doi:10.1371/journal.pone.0213608)
Supplement: S5 Table — Patients with DGF and no DGF show a comparable eGFR at all time points. P-values are from Mann-Whitney U tests. (DOCX) [file pone.0213608.s006.docx]

**Supporting information**

|  | **No DGF** | **DGF** | **p-value** |
| --- | --- | --- | --- |
| **3 months** | 52.5 (36.7, 70.0) | 46.2 (36.7, 54.1) | 0.095 |
| **1 year** | 53.1 (36.3, 73.4) | 51.3 (45.5, 58.2) | 0.740 |
| **3 years** | 52.1 (35.2, 70.5) | 56.0 (44.0, 69.7) | 0.576 |

**S5 Table. eGFR (CKD-EPI, ml/min/1.73m2, median (1st, 3rd quartile)) at three months, one and three years post RTx in recipients without AKI.** Patients with DGF and no DGF show a comparable eGFR at all time points. P-values are from Mann-Whitney U tests.
